# From a Single Penile Suspensory Ligament to a Penile Suspensory Apparatus: Application to the Buried Penis Surgery

**DOI:** 10.1093/asjof/ojad027.020

**Published:** 2023-04-14

**Authors:** Romain Laurent, Ruben Pierre Danino, Pierre Trouilloud, Mehdi Benkhadra, Michel Alain Danino

**Affiliations:** University Hospital of Montreal, Montreal, QC, Canada; McGill University, Montreal, QC, Canada; Faculty of Medicine of Dijon, Dijon, France; Faculty of Medicine of Dijon, Dijon, France; University Hospital of Montreal, Montreal, QC, Canada

## Abstract

**Goals/Purpose:**

The ligamentous system supporting the penis is widely called « penile suspensory ligament ». It supports and attaches the penis to the abdominal wall and the pubic bone. However, the complexity of the anatomy of the penile suspensory system was underestimated. The aim of this anatomical study was to describe the different ligamentous (features, insertion sites, path) and their functions.

**Methods/Technique:**

The first step consisted in a description of the different components of the penile suspensory apparatus on two cadavers embalmed by the Thiel method. The second part was an evaluation of the function of each ligament on freshly dead cadavers. In the third stage, we applied those findings to an exploratory cohort of patients undergoing a penile lengthening surgery for buried penis.

**Results/Complications:**

The penile suspensory apparatus consisted in four ligaments from anterior to posterior : the fundiform ligament, the suspensory ligament, dense vertical ligament and the arcuate ligament. The first two were loose, non-stabilizing and the two posterior ones were dense and stabilizing. In our cohort, 12 patients were included. The mean BMI was 38. The mean pre-operative flaccid length was 4.1 cm [2.2 cm – 7 cm] and at 6 months 8.2 cm [5.2 cm - 11.5 cm] which represents a mean augmentation of 100%. The mean pre-operative circumference at a flaccid state was 8.2 cm [2 cm – 11.5 cm] and at 6 months post-operative was 12.6 cm [8.3 cm - 16 cm] which represents a mean augmentation of 85.7%. The most common complication was a phimosis.

**Conclusion:**

We have described the detailed anatomy of the penile suspensory apparatus. It is the first time that the dense vertical ligament was brought to light. To avoid instability in penile lengthening, the posterior part of the dense vertical ligament and the arcuate ligament must be spared.

